# Supplementary material for: Dynamic protein phosphorylation shapes mitochondrial catalysis, import, and architecture
Source: bioRxiv. 2026 Sep 29:2026.09.28.755151. Preprint. [Version 1] doi: 10.64898/2026.09.28.755151 (PMC13648597; doi:10.64898/2026.09.28.755151)
Supplement: Supplementary file 1 [file NIHPP2026.09.28.755151v1-supplement-1.pdf]

bioRxiv preprint doi: https://doi.org/10.64898/2026.09.28.755151; this version posted September 29, 2026. The copyright holder for this preprint (which was not certified by peer review) is the author/funder, who has granted bioRxiv a license to display the preprint in perpetuity. It is made available under a CC-BY 4.0 International license.

SUPPLEMENTAL INFORMATION

All data produced in the present study are available upon reasonable request to the authors.

- Table S1. Phosphoproteomic and proteomic datasets of phosphatase CRISPRi experiment, related to Figs. 1, 2, 3, and 4.
- Table S2. Phosphoproteomic and proteomic datasets of PGAM5 rescue experiment, related to Fig. 4.
- Table S3. Proteomic datasets of MIC19 and ATP5I rescue cell lines, related to Fig S4.
- Table S4. Complexome profiling dataset of control and PGAM5-KD mitochondria, related to Fig. 5.
- Table S5. Proteomic dataset of MIC60 affinity enrichment experiment, related to Fig. 5.

bioRxiv preprint doi: https://doi.org/10.64898/2026.09.28.755151; this version posted September 29, 2026. The copyright holder for this preprint (which was not certified by peer review) is the author/funder, who has granted bioRxiv a license to display the preprint in perpetuity. It is made available under a CC-BY 4.0 International license.

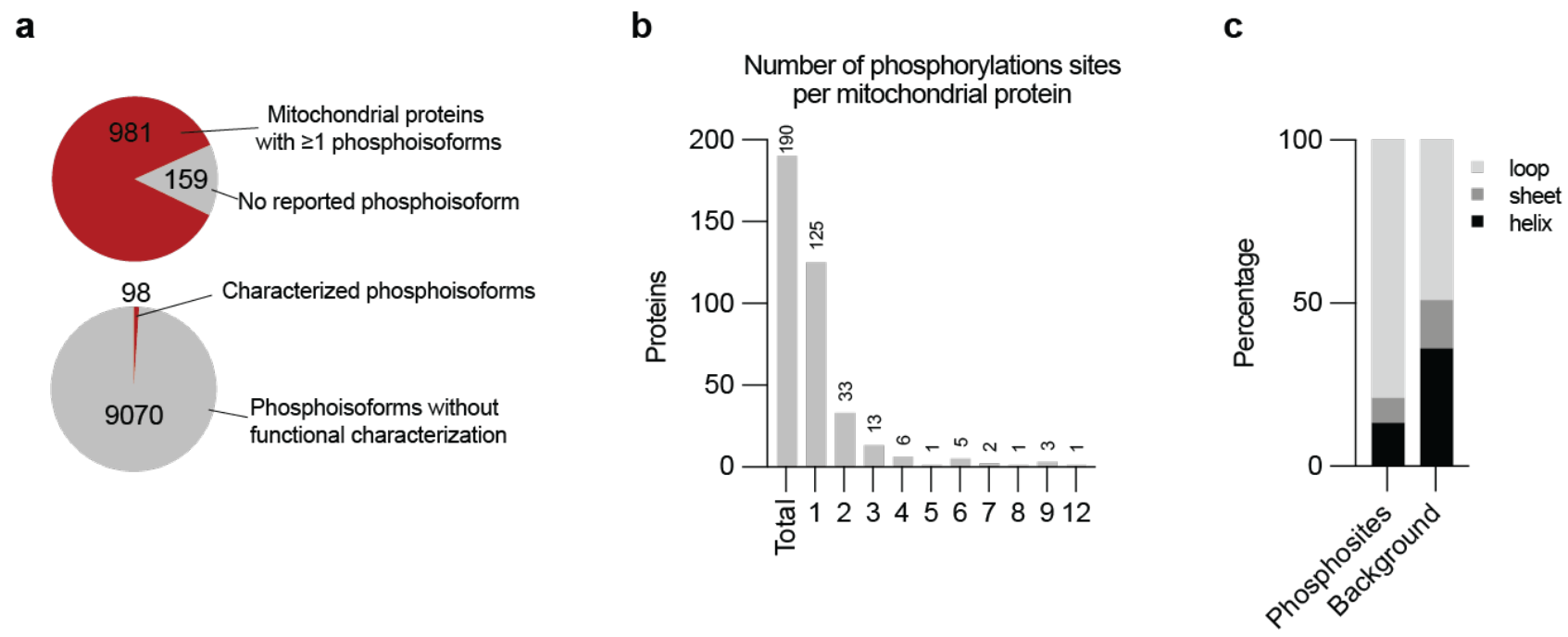

Figure S1.
(A) Fraction of MitoCarta3.0 proteins with at least one detected phosphoisoform (981) versus none (159) (top), and fraction of detected phosphoisoforms that are functionally characterized (98) versus uncharacterized (9,070) (bottom). Characterized sites curated from PhosphoSitePlus, mapped onto the MitoCarta3.0 mouse mitochondrial proteome. Known phosphosites from dbPTM (experimental catalog filtered to MitoCarta accessions) and EPSD (Mus musculus).
(B) Number of phosphorylation sites per mitochondrial protein (n = 190 phosphorylated proteins; median = 1).
(C) Secondary-structure composition (loop, sheet, helix) of phosphosites versus all other S/T/Y residues in the same proteins as background.

bioRxiv preprint doi: https://doi.org/10.64898/2026.09.28.755151; this version posted September 29, 2026. The copyright holder for this preprint (which was not certified by peer review) is the author/funder, who has granted bioRxiv a license to display the preprint in perpetuity. It is made available under a CC-BY 4.0 International license.

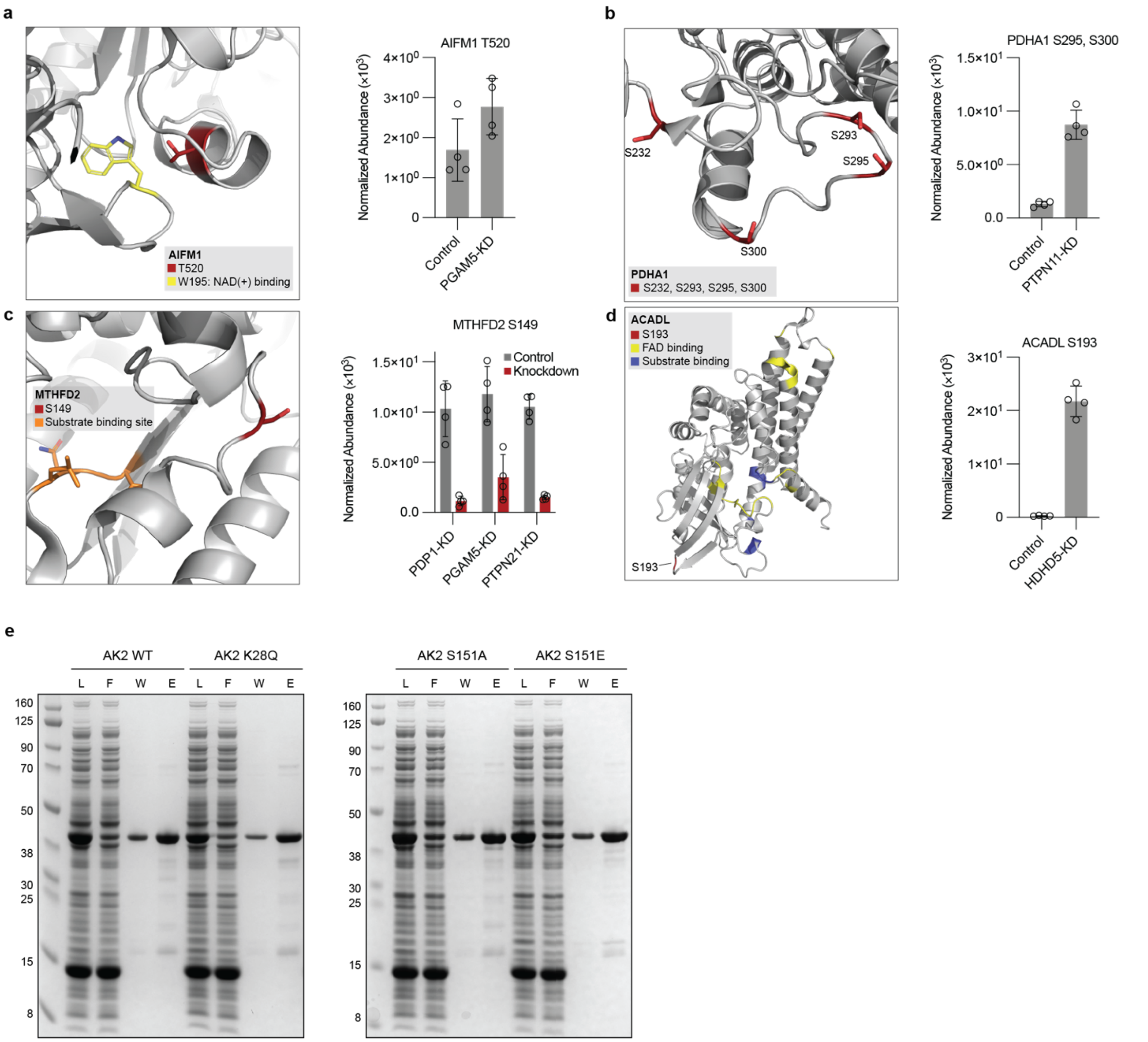

Figure S2.
(A) T520 in proximity to W195 in AIFM1, with protein normalized phosphopeptides abundance in PGAM5-KD.
(B) PDHA1 S232, S293, S295, and S300 mapped onto protein structure. PDHA1 S295, S300 protein-normalized phosphopeptide abundance in PTPN11-KD.
(C) S149 of MTHFD2 mapped in proximity to substrate binding site residues. MTHFD2 S149 protein-normalized phosphopeptide abundance in control and knockdown lines.
(D) ACADL S193 in proximity to FAD and substrate binding sites. ACADL S193 protein-normalized phosphopeptide abundance in HDHD5-KD cells.
(E) Coomassie stained SDS-PAGE gel of purified recombinant SUMO-tagged AK2 (WT, K28Q, S151A, S151E). L = loading, F = flow-through, W = wash, E = elution.

bioRxiv preprint doi: https://doi.org/10.64898/2026.09.28.755151; this version posted September 29, 2026. The copyright holder for this preprint (which was not certified by peer review) is the author/funder, who has granted bioRxiv a license to display the preprint in perpetuity. It is made available under a CC-BY 4.0 International license.

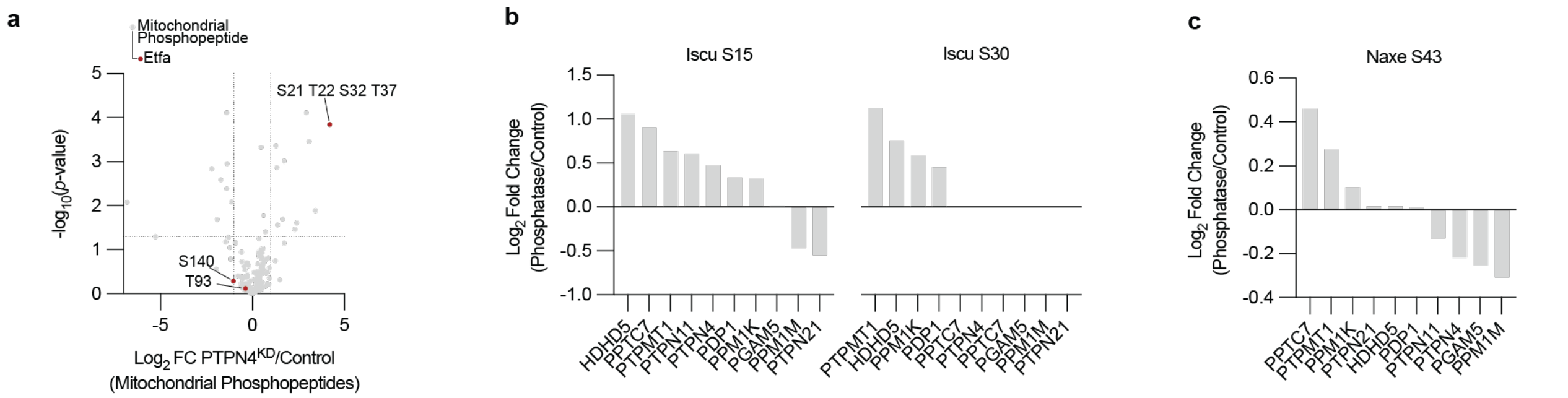

Fig. S3.

(A) Volcano plot of mitochondrial phosphopeptides in PTPN4-KD/Control cells showing significant elevation for a 4x phosphorylated Etfa peptide relative to two unchanged Etfa phosphopeptides.
(B) Iscu S15, and S30 log2 fold change of phosphatase knockdown lines compared to matched controls.
(C) Naxe S43 log2 fold change of phosphatase knockdown lines compared to matched controls.

bioRxiv preprint doi: https://doi.org/10.64898/2026.09.28.755151; this version posted September 29, 2026. The copyright holder for this preprint (which was not certified by peer review) is the author/funder, who has granted bioRxiv a license to display the preprint in perpetuity. It is made available under a CC-BY 4.0 International license.

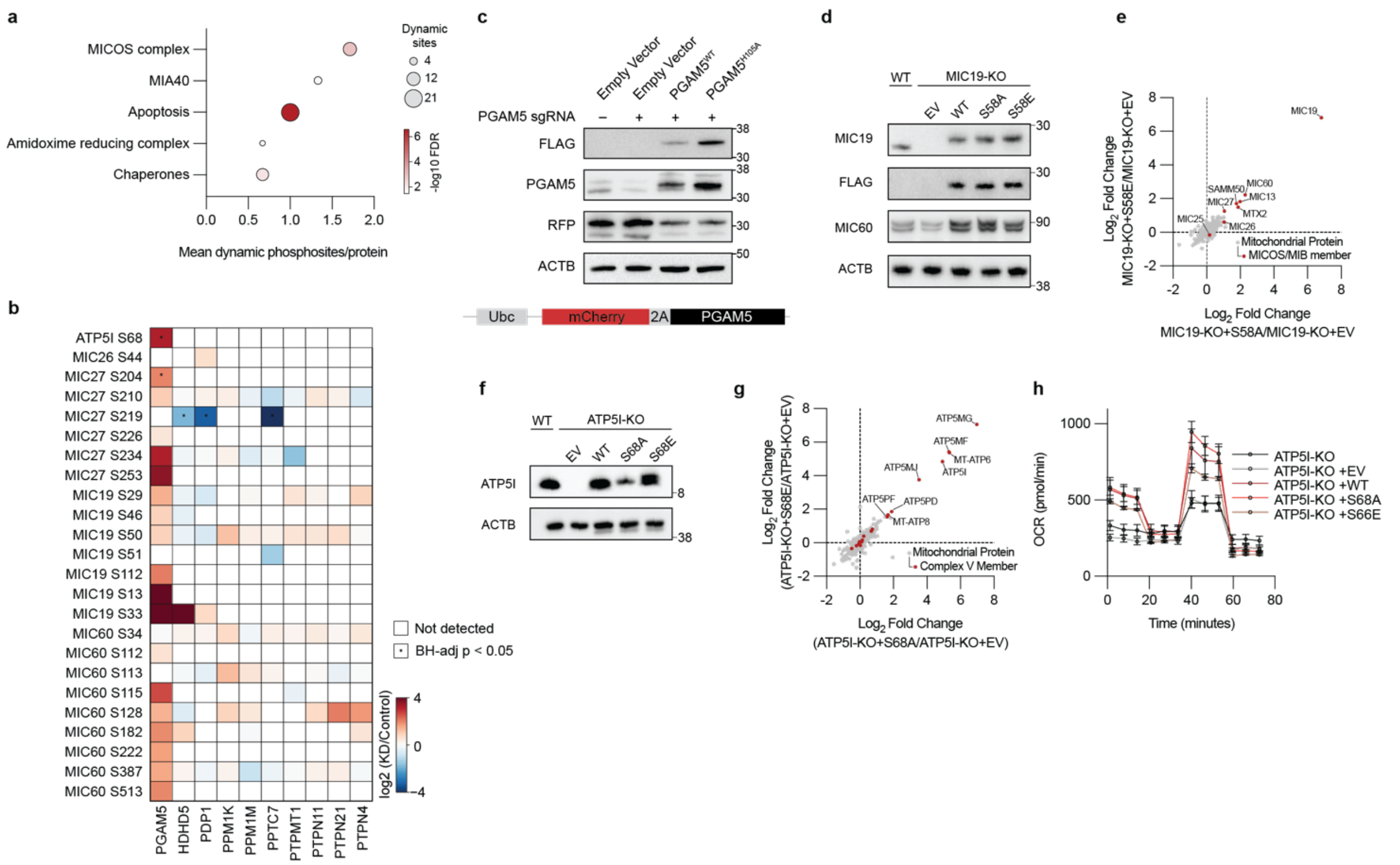

Figure S4.

(A) Phosphosite-weighted over-representation of MitoCarta pathways: each of the 828 detected mitochondrial proteins (union across the 10 per-line proteomes) is weighted by its number of dynamically regulated phosphosites (0 if none); pathway members compared to the rest of the proteome by one-sided Mann-Whitney U test, BH FDR.

(B) Log2 FC (KD/control) of all 23 quantified cristae formation phosphosites (ATP5I, MIC60, MIC19, MIC25, MIC27, MIC26) across all 10 phosphatase-knockdown lines. (Welch t-test, n = 4 SILAC replicates per line).

(C) Rescue construct schematic (ubiquitin promoter–mCherry-2A-PGAM5) and immunoblots confirming PGAM5 knock-down and WT/H105A re-expression (PGAM5 and C-terminal FLAG).

(D) Immunoblots of MIC19-KO cells reconstituted with WT, S58A, or S58E (MIC19, FLAG, MIC60, ACTB).

(E) Proteomic scatter, MIC19-KO+S58A/ vs MIC19-KO+S58E (log2 fold change over MIC19-KO+EV). Welch t-test, BH adjusted p-value; n = 3.

(F) Immunoblots of ATP5I-KO cells reconstituted with WT, S68A, or S68E (ATP5I, ACTB).

(G) Proteomic scatter, ATP5I-KO+S68A/EV vs ATP5I-KO+S68E/EV; defects restored by both. Welch t-test, BH adjusted p-value; n = 3.

(H) Seahorse OCR (pmol/min) of ATP5I-KO and rescues (EV, WT, S68A, S68E) in glucose.

bioRxiv preprint doi: https://doi.org/10.64898/2026.09.28.755151; this version posted September 29, 2026. The copyright holder for this preprint (which was not certified by peer review) is the author/funder, who has granted bioRxiv a license to display the preprint in perpetuity. It is made available under a CC-BY 4.0 International license.

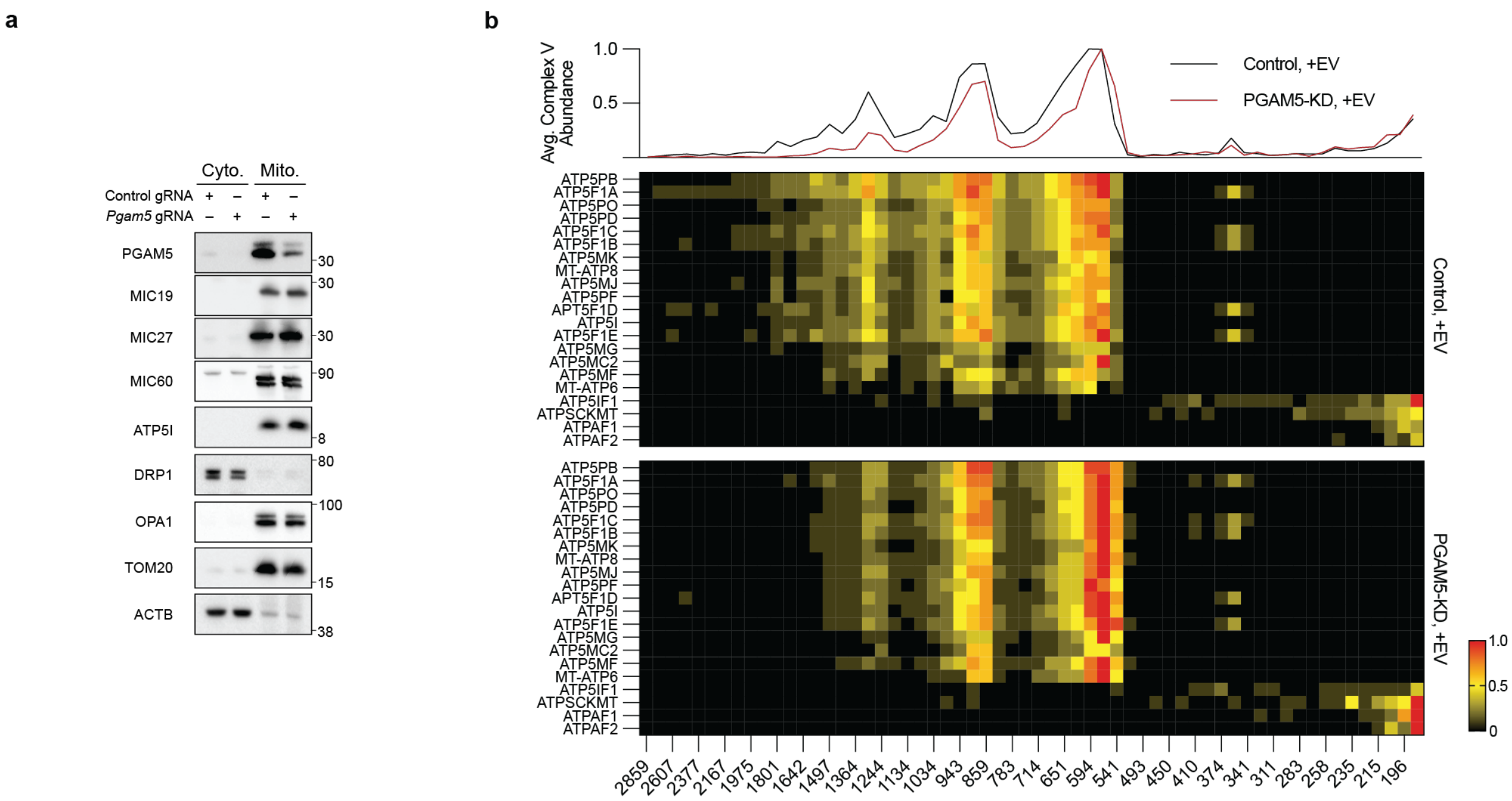

Figure S5.
(A) Immunoblots of purified mitochondrial and cytosolic fractions (control, PGAM5-KD) for PGAM5, MIC19, MIC27, MIC60, ATP5I, DRP1, OPA1. TOM20 and ACTB were used as mitochondrial and cytosolic loading controls.
(B) Complexome profiling heatmap and line profile of complex V (control vs PGAM5-KD); reduced dimeric/oligomeric-to-monomeric ratio in PGAM5-KD.
